# Supplementary material for: The role of social norms on adolescent family planning in rural Kilifi county, Kenya
Source: PLoS One. 2023 Feb 2;18(2):e0275824. doi: 10.1371/journal.pone.0275824 (PMC9894424; doi:10.1371/journal.pone.0275824)
Supplement: S1 File — (DOCX) [file pone.0275824.s001.docx]

Supporting Information

**Table S1.** Key variables and corresponding questionnaire items.

| Variable | Question Item(s) | Response Options |
| --- | --- | --- |
| Individual MC Use | “Are you or your partner currently using medical methods of family planning to delay or avoid having a child? (Remember, by medical methods of family planning we mean injectables, implants, the pill, condoms, the IUD, and sterilization).” | Yes/No |
| Network MC Use | Derived Item. Row mean of referents' (alters') response to the individual MC Use question. |  |
| Perceived Descriptive Norms | 1. “Most people around me use medical methods of family planning for determining when to have a child.” 2. “Most people whose opinions I value use medical methods of family planning for determining if or when to have a child.” | Strongly Disagree, Disagree, Neutral, Agree, Strongly Agree |
| Perceived Injunctive Norms | 1. “Most people important to me will think badly of me if I use medical methods of family planning.” 2. “Most people important to me will reject me if I use medical methods of family planning.” | Strongly Agree, Agree, Neutral, Disagree, Strongly Disagree |
| MC Use Leads to Health Problems (MC Belief #1) | Using medical methods of family planning can cause health problems. | Strongly Agree, Agree, Neutral, Disagree, Strongly Disagree |
| MC Use Leads to Lost trust between partners (MC Belief #2) | Using medical methods of family planning can cause a loss of trust between a person and his/her partner. | Strongly Agree, Agree, Neutral, Disagree, Strongly Disagree |
| Sexual activity is ok (MC Attitude #1) | It is OK for adolescents to be sexually active. | Strongly Disagree, Disagree, Neutral, Agree, Strongly Agree |
| MC Use is ok (MC Attitude #2) | If adolescents are sexually active, it is OK for them to use medical methods of family planning. | Strongly Disagree, Disagree, Neutral, Agree, Strongly Agree |
| Reference Group Identification | With whom did you spend a lot of free time in the past year?  (Probe: You can name anyone who is 15 years old or above and who currently lives or works in this village. This person could be your family members (partner, parent, relative), neighbors, health workers (doctor, nurse, CHV), friends, someone at school, church, or community organization, etc. We will provide you with a list of these categories to remind you. This person could be someone you talk with while fetching water, going to church, or chatting in front of your house as recently as the past week or anytime in the past year.) | Name and Relationship (up to 5) |

**Table S2**. Bivariate and multivariable regression models from sensitivity analysis.

|  | Individual MC Use (N = 759) | | | | | |
| --- | --- | --- | --- | --- | --- | --- |
|  | OR (95% CI) | | | Adjusted OR (95% CI) | | |
|  | (1) | (2) | (3) | (4) | (5) | (6) |
|  | | | | | | |
| Network MC Use | 2.66^***^  (1.68,4.19) | 1.56  (0.92,2.64) | 1.55  (0.91,2.61) | 1.53  (0.9,2.61) | 1.52  (0.89,2.59) | 1.50  (0.87,2.57) |
| Perceived Descriptive Norms | 1.27^**^  (1.06,1.51) | 1.29^*^  (1.04,1.59) | 1.25^*^  (1.01,1.55) | 1.29^*^  (1.04,1.59) | 1.28^*^  (1.04,1.59) | 1.26^*^  (1.02,1.56) |
| Perceived Injunctive Norms | 1.54^***^  (1.28,1.84) | 1.32^**^  (1.07,1.63) | 1.26^*^  (1.02,1.56) | 1.32^**^  (1.07,1.63) | 1.32^**^  (1.07,1.63) | 1.27^*^  (1.02,1.57) |
| Perceived Injunctive Norms x Perceived Descriptive Norms |  |  | 1.19  (0.97,1.47) |  |  | 1.18  (0.96,1.46) |
| Network MC Use x Perceived Descriptive Norms |  |  |  | 1.14  (0.62,2.09) |  | 1.11  (0.6,2.03) |
| Network MC Use x Perceived Injunctive Norms |  |  |  |  | 1.18  (0.68,2.05) | 1.12  (0.64,1.96) |
| Age | 1.17^***^  (1.1,1.24) | 1.10^*^  (1.02,1.19) | 1.11^*^  (1.02,1.2) | 1.10^*^  (1.02,1.19) | 1.10^*^  (1.02,1.19) | 1.11^*^  (1.02,1.2) |
| Married [Ref: Unmarried] | 2.52^***^  (1.78,3.56) | 2.58^***^  (1.54,4.31) | 2.51^***^  (1.5,4.21) | 2.56^***^  (1.53,4.29) | 2.61^***^  (1.56,4.37) | 2.52^***^  (1.5,4.24) |
| *Education* [Ref: No Schooling] |  |  |  |  |  |  |
| Primary School | 1.11  (0.47,2.62) | 2.00  (0.79,5.05) | 1.86  (0.73,4.72) | 2.00  (0.79,5.05) | 2.02  (0.8,5.11) | 1.87  (0.73,4.79) |
| Post-Primary/Vocational/Other | 1.39  (0.52,3.71) | 2.50  (0.83,7.55) | 2.35  (0.77,7.16) | 2.51  (0.83,7.57) | 2.51  (0.83,7.61) | 2.37  (0.78,7.24) |
| Secondary/'A' level | 0.70  (0.29,1.68) | 1.69  (0.63,4.58) | 1.56  (0.57,4.26) | 1.69  (0.63,4.58) | 1.71  (0.63,4.64) | 1.58  (0.58,4.33) |
| College/University | 0.87  (0.3,2.56) | 1.21  (0.36,4.1) | 1.12  (0.33,3.81) | 1.21  (0.36,4.1) | 1.23  (0.36,4.16) | 1.13  (0.33,3.87) |
| Lower MC Use Village [Ref: Higher MC Use Village] | 0.60^**^  (0.43,0.85) | 0.98  (0.63,1.52) | 0.99  (0.64,1.54) | 0.98  (0.63,1.51) | 0.98  (0.63,1.52) | 0.99  (0.64,1.53) |
| Female [Ref: Male] | 0.60^**^  (0.43,0.82) | 0.51^**^  (0.34,0.78) | 0.51^**^  (0.34,0.78) | 0.51^**^  (0.34,0.78) | 0.51^**^  (0.33,0.77) | 0.51^**^  (0.34,0.78) |
| MC Use leads to health problems | 1.32^***^  (1.13,1.53) | 1.12  (0.93,1.35) | 1.11  (0.91,1.34) | 1.12  (0.93,1.35) | 1.12  (0.92,1.35) | 1.10  (0.91,1.33) |
| MC Use leads to lost trust between partners | 1.21^*^  (1.03,1.41) | 1.10  (0.92,1.32) | 1.10  (0.91,1.32) | 1.10  (0.91,1.32) | 1.10  (0.91,1.32) | 1.09  (0.91,1.32) |
| Sexual activity is ok | 1.84^***^  (1.56,2.15) | 1.56^***^  (1.3,1.88) | 1.57^***^  (1.3,1.89) | 1.57^***^  (1.3,1.89) | 1.57^***^  (1.3,1.89) | 1.57^***^  (1.3,1.89) |
| MC use is ok | 1.48^***^  (1.3,1.68) | 1.34^***^  (1.15,1.56) | 1.36^***^  (1.17,1.58) | 1.34^***^  (1.15,1.55) | 1.34^***^  (1.15,1.56) | 1.35^***^  (1.16,1.58) |
| Muslim [Ref: Other] | 1.36  (0.99,1.87) | 1.03  (0.69,1.55) | 1.06  (0.7,1.59) | 1.04  (0.69,1.56) | 1.03  (0.69,1.55) | 1.06 (0.71,1.6) |
|  | | | | | | |
| McFadden’s Pseudo $\boldsymbol{R}^{\boldsymbol{2}}$ |  | 0.173 | 0.176 | 0.173 | 0.173 | 0.176 |
| Akaike Information Criterion |  | 788.30 | 787.58 | 790.13 | 789.95 | 791.29 |
| *^*^p<.05 ^**^p<.01 ^***^p<0.001* | | | | | | |
|  | | | | | | |

Note: continuous variables are mean-centered.

**Table S3.** Stratified Regression Results from sensitivity analysis.

|  | Individual MC Use  Adjusted OR (95% CI) | | | |
| --- | --- | --- | --- | --- |
|  | Females  (N = 436) | Males  (N =323) | Married Females  (N = 164) | Unmarried Females  (N = 272) |
|  | (1) | (2) | (3) | (4) |
|  | | | | |
| Network MC Use | 2.75^**^  (1.33,5.69) | 0.71  (0.31,1.61) | 1.88  (0.71,4.98) | 7.32^**^  (2.02,26.57) |
| Perceived Descriptive Norms | 1.33  (0.95,1.86) | 1.28  (0.96,1.72) | 1.13  (0.7,1.83) | 1.36  (0.77,2.41) |
| Perceived Injunctive Norms | 1.25  (0.94,1.67) | 1.40^*^  (1.02,1.94) | 1.30  (0.86,1.94) | 1.09  (0.68,1.74) |
| Age | 1.19^**^  (1.07,1.33) | 1.02  (0.91,1.15) | 1.13  (0.96,1.33) | 1.33^***^  (1.13,1.57) |
| Married [Ref: Unmarried] | 2.35^*^  (1.21,4.57) | 1.44  (0.53,3.94) |  |  |
| *Education* [Ref: Primary School] |  |  |  |  |
| No Schooling | 0.33^*^  (0.12,0.93) | 2.12  (0.11,41.25) | 0.40  (0.14,1.17) |  |
| Post-Primary/Vocational/Other | 1.32  (0.55,3.2) | 1.43  (0.49,4.18) | 0.86  (0.22,3.44) | 1.35  (0.41,4.48) |
| Secondary/'A' level | 0.50  (0.23,1.08) | 1.21  (0.68,2.14) | 0.61  (0.12,3.02) | 0.52  (0.2,1.38) |
| College/University | 0.09^*^  (0.01,0.79) | 1.55  (0.52,4.63) | 1.11  (0.08,14.66) |  |
| Lower MC Use Village [Ref: Higher MC Use Village] | 0.65  (0.33,1.24) | 1.27  (0.67,2.4) | 0.27^**^  (0.11,0.69) | 1.88  (0.66,5.4) |
| MC Use leads to health problems | 0.99  (0.75,1.31) | 1.16  (0.88,1.52) | 1.18  (0.82,1.7) | 0.70  (0.4,1.22) |
| MC Use leads to lost trust between partners | 1.20  (0.91,1.57) | 1.02  (0.77,1.33) | 1.22  (0.84,1.77) | 1.36  (0.86,2.16) |
| Sexual activity is ok | 1.20  (0.86,1.66) | 1.85^***^  (1.45,2.36) | 0.99  (0.62,1.58) | 1.40  (0.87,2.25) |
| MC use is ok | 1.29^*^  (1.05,1.59) | 1.44^**^  (1.15,1.8) | 1.02  (0.75,1.37) | 1.76^**^  (1.23,2.52) |
| Muslim [Ref: Other] | 0.91  (0.51,1.64) | 1.04  (0.57,1.89) | 1.02  (0.46,2.27) | 1.23 (0.45,3.34) |
|  | | | | |
| *^*^p<0.05 ^**^p<0.01 ^***^p<0.001* | | | | |
|  | | | | |

Note: continuous variables are not mean-centered.
