# Supplementary material for: The role of social norms on adolescent family planning in rural Kilifi county, Kenya
Source: PLoS One. 2023 Feb 2;18(2):e0275824. doi: 10.1371/journal.pone.0275824 (PMC9894424; doi:10.1371/journal.pone.0275824)
Supplement: Attachment — Submitted filename: Response to Reviewers.docx [file pone.0275824.s002.docx]

**Response to Reviewers**

April 15, 2022

Thank you for the opportunity to revise and resubmit our manuscript. We believe that the manuscript is stronger for having addressed the thoughtful feedback from the reviewers. The following is a summary of responses to each comment from the reviewers and from PLOS ONE. Comments are in Black, and our responses are in Red.

## Comments from PLOS ONE

1. Please ensure that your manuscript meets PLOS ONE's style requirements, including those for file naming.

We have followed PLOS ONE’s style requirements, including those for file naming. This includes inserting line numbers, adding the title page to the manuscript, and following the file naming guidelines.

2. Please ensure that you include a title page within your main document. We do appreciate that you have a title page document uploaded as a separate file, however, as per our author guidelines (http://journals.plos.org/plosone/s/submission-guidelines#loc-title-page) we do require this to be part of the manuscript file itself and not uploaded separately.
Could you therefore please include the title page into the beginning of your manuscript file itself, listing all authors and affiliations.

We have included the title page at the beginning of the manuscript, with all authors and affiliations.

3. Please include your full ethics statement in the ‘Methods’ section of your manuscript file. In your statement, please include the full name of the IRB or ethics committee who approved or waived your study, as well as whether or not you obtained informed written or verbal consent. If consent was waived for your study, please include this information in your statement as well.

We have included a more complete ethics statement at the end of the Data and Study Design section in ‘Methods. We have noted the full name of the IRB which approved this study, the approval ID, and an explicit mention of informed oral consent obtained from all study participants.

## Comments from Reviewer #1

Methods
- Can you provide a bit more detail about the original survey used to identify the two villages with lower and higher MC use? Were the two estimates of MC use (10.3% and 44.4%) derived from an adolescent population, or did the sample for those figures include adults as well?

We have now clarified that that the estimates of MC use in the villages were derived from women of childbearing age (15-49 years). We have also clarified the data collection year (2018), and added a few more details about the process.

- The Data and Study Design subsection would benefit from additional information on data collection and ethical procedures:

o Were both informed parental consent and respondent assent obtained for those under 18?

Yes, we have now clarified this in the first paragraph of the “Data and Study Design” section. Individuals 18 years or older provided informed oral consent. Individuals between 15 and 17 years provided informed assent, and for them we also obtained parental consent.

o Please include the IRB approval information

IRB approval information has now been included.

o Did you have a target sample size? Please add sample size calculations

We have now clarified in paragraph 2 of the “Data and Study Design” section that the aim was to survey 100% of the eligible men and women for the study, but the overall response rate was 72%. There were no other sample size calculations or considerations.

Results

- Table 2 finds MC use to be 33% and 23% in the high and low MC use villages, respectively. Do you have a sense of why these numbers differ from the initial estimates used to select the villages (44.4% and 10.3%)? Is it because the initial estimates were derived from an adult- and not adolescent- population (this question relates to my first comment for the Methods section)?

This is exactly right. The discrepancy is because the larger figures are for women of reproductive age (15-49), and the results in table 2 reflect the MC use for our sample (15-24). We are hopeful this is now evident from our clarification of this point in the Methods section.

- Apologies if I missed this somewhere in the article, but I think it would be helpful to see whether the descriptive and injunctive norms are correlated with network use. This would help to confirm whether the individuals identified as members of one’s network are comparable to “those around me” and “those close to me,” as framed in the norms questions.

None of the correlations between network MC Use, descriptive norms, and injunctive norms were higher than 0.1, nor were they significant. We have now added two sentences after Table 1 to clarify this.

## Comments from Reviewer #2

Reviewer #2: While the topic of the paper is important, the data are unique, and the methods seem appropriate, I find the paper is written more as a report with insufficient detail and thought spent on the type of presentation that I would expect for a publication in a peer-reviewed paper. I sincerely hope that the authors re-write the paper with more care – as I think the potential is there for a very interesting paper. It is for this reason that I include the following specific comments that I hope the authors find useful.

Abstract

Overall, I find the abstract somewhat impenetrable, even for someone familiar with the approach and topic. The Purpose section is mostly methods and is missing the motivation for the research. The Methods section has insufficient details about the approach. The Results section should include some key statistics with information on the directionality of the effects. The Conclusions emphasize the findings for unmarried women, whom I assume are those “living with a partner” in this study, but the authors never make that clear. I suggest using one term or the other throughout the paper – but make it clear that unmarried doesn’t mean single. Not all readers will be familiar with the term “sociometric.”

We have rewritten the abstract entirely. The Purpose section now contains background information and motivation for the study. The Methods section now has much greater detail on data collection, variable creation, and analysis approach. The Results section now presents specific statistics with directionality to illustrate key results. The Conclusions section emphasizes the findings for unmarried women living with a partner. We have also clarified the “living with a partner” criterion in the Methods section of the manuscript. We have removed the term ‘sociometric’ in the Abstract, and written the methods more descriptively.

Introduction

1. The first sentence about findings in 2020 suggests that family planning use has improved substantially from 2014 in Kenya, making it difficult for the reader to know whether they should be concerned about the 2014 statistics presented in the same paragraph. Suggest updating the 2014 statistics or re-contextualizing the information.

We have updated the statistics using data from Q1 of 2021, as reported by the Performance Monitoring and Accountability (PMA) project. We have updated a few different parts of the Introduction to reflect more recent data and findings, including how COVID-19 has impacted the rate of teen pregnancy and school dropout among girls in Kenya.

2. The authors should familiarize themselves with the following reference on social norms and adolescent pregnancy in Honduras (Shakya HB, Darmstadt GL, Barker KM, Weeks J, Christakis N. Social normative and social network factors associated with adolescent pregnancy: a cross-sectional study of 176 villages in rural Honduras. J Glob Health 2020;10:010706.)

Thank you for sharing this reference. The study by Shakya and colleagues is highly relevant to our own, and shares a similar focus on norms, and a similar methodology. We have cited this study in the first sentence of the Discussion section, as we found it fit better in that section.

3. I would expect that the aims for the research would be included in paragraph form at the end of the introduction. The presentation format for the research questions and hypotheses strikes me as a style reserved for protocols or grant proposals.

We have removed the “Research Questions and Hypotheses” section. Instead, we have described our hypotheses in narrative form in the last paragraph of the Introduction section, as recommended.

4. Regarding the research questions, I am surprised that the stratification by males/females and married/unmarried were not represented. Presumably the researchers hypothesized that there would be differences by sex and marital status given sexual double standards and gender norms in the region. If so, why not include questions to investigate these differences?

Indeed, this was the main motivation for our stratified analyses. We have now made this explicit in the last paragraph of the Introduction section, when discussing our aims and hypotheses.

Methods

1. The authors should explain their motivation for selecting villages with a high and low prevalence MC use (e.g., to assure that they had sufficient variation in responses, perhaps?). On a related note, why did they not look at the associations stratified by village, for example, asking, whether the associations of norms with individual MC use differed in the high vs. low prevalence MC use villages?

We have now explained the purpose of selecting both villages, as they represent different stages of fertility transition, in the Methods section. We chose to look only at the descriptives by village, rather than stratifying the main analyses by village, primarily due to sample size. Once we break down the sample by village, and then to further stratify on gender and marital status, would drastically reduce our statistical power to detect our relationships of interest. Given that our focus was to determine the structure of normative relationships with MC use, we opted to use a pooled sample for the regressions.

2. How many people were dropped because they did not nominate a referent?

Thirty-two individuals were dropped for not nominating a referent in the main regression models. We have now clarified this point in the second paragraph of the Methods section.

3. For the “aggregation” of two items – were these at the individual level? Is so, it would be less confusing to simply state that they were averaged. The authors refer to “aggregating” individual behaviors to enumeration areas.

We have removed “aggregate” and its variations when describing variable creation for our study. Instead, we have simply written “by averaging two items for each variable.”

4. The authors should be clear that the perceived injunctive norms were recoded, similar to the MC beliefs. Related to this – the wording for MC beliefs is negative in the tables, but I think that the finding is that “MC use does NOT lead to health problems” is associated with increased MC use. I suggest changing the table text by clarifying the reference category.

Actually, the wording in the tables is correct, and it is the response options which indicate that variables are coded such that higher values are more pro-MC. To change the wording in the tables for the MC beliefs would not reflect how the questions were asked, and would actually be the reverse of the way the variables were coded. However, we have attempted to clarify this by adding an example and making clear the direction of the response options in the last two paragraphs of the Methods section.

Results

1. The results are missing an introductory paragraph describing the sample numbers, response rates, and missing data information. Also, the description of results included in the text needs more detail about comparison groups (e.g., for females as compared to males) and the directionality of the association (e.g., increasing individual use with increasing age).

We have added an introductory paragraph describing the sample characteristics in Table 1. Additionally, we have also added Network MC Use (with n_missing) and perceived norms to Table 1.

2. As the paper stands, I don’t find the stratification of the descriptive data in Table 2 very useful. It would be more interesting to see these data by sex and marital status. However, I am curious to know how different the injunctive norms are by village type.

We have changed Table 2 so that it is now stratified by marital status, which we believe is more useful than stratifying by village or sex. We have also now included all the norms variables in Table 1, so they can be compared across villages.

3. Tables with regression results should include either SEs or CIs – my preference would be to include upper and lower 95% CIs.

We have now added 95% CI for all coefficients in all regression tables, including those in the supplementary file.

4. Were other potential confounding variables considered, such as labor force participation, household or village wealth, or village size or distance to roads? If not, why not?

In general, our approach was to select a parsimonious, yet fairly representative, set of confounders to avoid overfitting the models. Village-level covariates such as size, distance to roads, and others are captured by including the village as a covariate in all models. We did not include individual income or labor force participation for two reasons. For one, the adolescent sample was young and was not characterized by high labor force participation. To account for household wealth, we selected education as a proxy, as this particular dataset did not contain an income variable. We believe that including village, age, and education, in addition to the other control variables, serve as a reasonable set of confounders.

Discussion

1. Overall, I find the discussion weak with repetition of text from the results, little reference to how the findings fit in with what has been shown in the literature, and no discussion of broader policy or program planning implications.

We have largely rewritten the Discussion section. The first three paragraphs are new content to situate the findings within the broader literature on social norms around MC use in Kenya, and also incorporates recent evidence on COVID-19 and FP decisions, and legal protections around MC use for adolescents. We have also added recommendations for measuring social norms which are absent from existing large-scale surveys like the DHS and PMA. We believe the Discussion section now addresses broader implications for programs, policy, and research in a more substantive manner.

2. I don’t think the findings from the sensitivity analyses about unmarried females should be emphasized in the discussion as a main finding, unless they are moved to the main paper.

We have removed these findings from the sensitivity analyses in the Discussion section.

3. There are a couple of comments about findings that are not presented in the text and therefore should either not be in the discussion or be added to the results:
a. “…when unmarried women come from social networks in which others (particularly same-sex others) are using contraceptives…” Results aren’t presented by whether the alter is same-sex or not (unless I missed them?).
b. “That this study was still able to detect some interactions between norms variables in the stratified analyses…” I didn’t see any results of interactions in the stratified analyses.

We have rewritten the paragraph with these findings. For point a) we have clarified that the relationship between network MC use and individual MC use was stronger for females than for males. Further, this relationship was stronger for unmarried females compared to married females. Given that same-sex referents were most commonly nominated by female participants, we suggest that future research investigate whether the stigma around MC use among unmarried females can be blunted by them affiliating with MC-using same-sex referents. For point b) we have removed this statement.

4. The sentence stating that “the omission of these individuals does not compromise our findings” is problematic since the omission could, in fact, result in selection bias that could result in significant but invalid findings. The authors should discuss how the missing observations are unlikely to lead to selection bias.

We have now clarified this. We concede that selection bias may be at play with our sample. It is not possible to rule this out. Rather, we suggest that the omission of these individuals does not compromise *the utility of our findings for future research* that seeks to clarify whether these patterns hold in larger samples. We are merely suggesting the social norms are an important influence on individual MC use, and that network MC use may be especially influential for female participants in particular.
