# Supplementary material for: The role of social norms on adolescent family planning in rural Kilifi county, Kenya
Source: PLoS One. 2023 Feb 2;18(2):e0275824. doi: 10.1371/journal.pone.0275824 (PMC9894424; doi:10.1371/journal.pone.0275824)
Supplement: Attachment — Submitted filename: Response to Reviewers.docx [file pone.0275824.s003.docx]

**Response to Reviewers**

June 17, 2022

Thank you for the opportunity to revise and resubmit our manuscript. We believe that the manuscript is stronger for having addressed the additional feedback from Reviewer #2. The following is a summary of responses to each comment from Reviewer #2. Comments are in Black, and our responses are in Red.

**Comments from Reviewer #2**

Reviewer #2: The paper has been much improved. However, a number of concerns remain.

**Abstract**
• Methods: Unclear what the authors mean by “aggregating the proportion”

This has been changed to “taking an average of an individual’s referents who use modern contraception.”

• Results: Please replace p-values with upper and lower 95% CIs for effect estimates

Done.

• Results: The “strength” of an association is determined by the magnitude of the coefficient not the p-value. Please edit accordingly.

This is a good point. We have made the corresponding changes.

• Results: Abbreviation MC was used without previous explanation (i.e., include (MC) after first use of modern contraception).

We have changed ‘MC’ to “modern contraceptive use” in the Abstract. In the manuscript, we have introduced ‘MC’ after the first instance of “modern contraception.”

**Introduction**
• Line 149-151 – isn’t the Shakya paper an example? Another reference that is relevant is: Mejía-Guevara I, Cislaghi B, Weber A, et al. Association of collective attitudes and contraceptive practice in nine sub-Saharan African countries. J Glob Health. 2020;10(1):010705. doi:10.7189/jogh.10.010705

### The study by Mejía-Guevara et al. (2020) uses individual attitude items, and aggregates them to a community level for a measure of “collective attitudinal norms.” Even though the underlying methodology (of aggregation to the collective from the individual level) is similar to ours, we hesitate to rely on this for two reasons. First, as the authors concede on pg 2, attitudes are not norms, and indeed the authors use them as proxies for norms. The divergence between personal attitudes and normative perceptions can be drastic (e.g. pluralistic ignorance) and thus we believe this measure can be a potentially noisy approximation for norms. Second, ours is a measure of behavioral prevalence within a reference group, rather than a measure of attitudinal prevalence. In order to keep the two separate, we did not rely on the Mejia-Guevara et al. study, but remain open to doing so if the reviewer feels strongly that we should.

We agree about using the Shakya study. Though they use a village-level reference group, rather than proximal reference group, to estimate collective norms, their inclusion of alters’ normative perceptions is conceptually similar to our approach. We have now included a sentence acknowledging the Shakya study in this section, and modified the concluding sentence of the paragraph to clarify that we are not aware of studies in the adolescent family planning literature in *sub-Saharan Africa* investigating the relationship between collective norms, operationalized as behavioral prevalence within a proximal social network, on individual behavior.

• Line 153 – Abbreviation MC was used without previous explanation (i.e., include (MC) after first use of modern contraception).

Abbreviation MC has now been placed after the first instance of the term “modern contraception” used in Sentence 2.

• Line 157 – Why do the authors hypothesize that there would be significant interaction between the 3 norm-related variables (other than the one example) in this particular context?

We have now clarified that these hypotheses stem from the Expanded Theory of Normative Social Behavior (Rimal and Yilma, 2021). This expansion suggests that collective, injunctive, and descriptive norms may operate differently for adolescent males and females, and that the interactions between normative variables can be used to understand different normative scenarios on behavior (e.g. scenarios where all norms are high, scenarios where all are low, and combinations of each).

**Methods**
• What do the authors mean by higher and lower “fertility transition” for the villages?

We have now clarified that different levels of fertility transition refer to the differential rate of decline from high to lower fertility between villages. The decline was sharper in one village as compared to the other.

• The content of the methods section is much better, but it needs re-organization for logical flow and additional clarity. For example, the section describing the sample is confusing with duplication of information. Referents are first described in the “data and study design” section but how they were nominated is in the subsequent section on measures. Similarly, information about the main outcome is first introduced the “data and study design” section but should be under measures with the first paragraph in this section.

We appreciate this suggestion, but believe that combining elements about how variables were created (from the ‘Measures’ section) with how sampling was conducted (from the “data and study design” section) would conflate the two. Rather, we prefer to explain the study design elements (such as sampling strategy and data collection process), and how the variables were created, in separate sections. Though these may appear redundant, they refer to different aspects of our study.

For example, the mention of referents in the “data and study design” section relates to the sampling design, in that trained enumerators matched egos with alters (referents) through photographs using the Trellis software. This is different from how referents are mentioned in the ‘Measures’ section, in which we created the network MC use variable by taking an average of referents’ MC use. The former refers to a data collection approach, while the latter refers to variable creation.

Similarly, if the reviewer is referring to the mention of high and low MC use villages in stating that the main outcome is introduced in the “data and study section,” this is again a question of sampling design vs measure creation. With regard to sampling, we describe how *village-level* MC prevalence was used a criterion for village selection. Distinctly, *individual* MC use was described as the main outcome in the ‘Measures’ section. The former refers to sampling and the latter refers to a different variable used for our statistical models. We hope the reviewer agrees about the underlying rationale – to keep separate the description of the study design from that of the measures.

• Which analyses were restricted to those married or living with a partner? Regression analyses? I see "single" in the descriptive statistics. Were singles (the majority of the sample) excluded from the regression models and why?

Thank you for catching that. We have removed the sentence stating that the analysis was restricted to those married or living with a partner. The analysis included those who were unmarried (single), who were not cohabiting with a partner, as well as those who were married or cohabiting with a partner.

• Failure to nominate a referent is a criterion for exclusion from the regression analyses, but was included in the descriptive statistics. Since missing for this and individual MC use are described in the results, I think the missing info on network MC use can be removed from the methods section to avoid duplication.

We have removed this information from the methods section.

• Try to keep the order of the norm-related variables the same throughout – e.g., - network MC use, descriptive norms, and perceived injunctive norms (the order they were presented in the methods).

We have now ensured the norms variables are presented in this order in the narrative and for the main effects in all tables.

**Results**
• Please clarify the sample size used in different analyses (descriptive vs. regressions). Include N’s in the tables, especially the stratified analyses.

The Ns are now present in all tables in the Headers.

• Why is the % of missing so high for Traditional Contraception Use among non-MC users in Table 2?

The denominator for the missing in Traditional Contraception Use among non-MC users is the full sample (n = 763), most of whom were MC users. For clarity, we have now removed this row.

• Consider showing one star for p-value <0.1, 2 stars for <0.05, and 3 stars for <0.01. There is little to be gained by indicating p-values <0.001 over <0.01 (this is not a measure of strength but of precision). My guess is that network MC use is significant at the 10% level in the full sample. Given that the magnitude and direction of effect is consistent with the other findings, I might argue that the adjusted effect is meaningful and the wide confidence intervals may be due to insufficient power or effect modification by sex (unless the authors think the positive association is biased after adjusting for confounders).

We discussed this issue internally and came to the conclusion that, as our alpha threshold of 0.05 was determined a priori, we should retain our star levels as is – with the conventional *for p<0.05, ** for p<0.01, and *** for p<0.001. This is also consistent with most published literature in our field. As we have included 95% CIs for coefficients, readers will be able to see if certain estimates have come close to the threshold. We hope the reviewer agrees with this decision.

• If, as the authors suggest in the discussion, the effect of network MC use on individual MC use is mediated through perceived norms (i.e., the perception variables are blocking part of the path), then why not include an initial model with the network variable but without the other 2 perception variables? While this approach doesn’t provide “strong” evidence of mediation – it would be suggestive and support further research.

We appreciate the suggestion to include a model without the perceived norms variables. We have been careful to include both explanatory and exploratory models we had specified a priori, and wish to avoid running additional models for this study. Further, three factors make us think that such a model might not be necessary: 1) it is likely that we may not have sufficient statistical power to detect a mediational effect, if it exists, 2) a significant network MC use coefficient in a model without perceived norms may reflect an inflated Type I error due to multiple hypothesis tests from additional models, and 3) our cross-sectional study design precludes the establishment of temporal precedence. Thus, we would not be able to argue convincingly that network MC use influences perceived norms, or the other way around. For these reasons, we believe a more robust treatment of mediation is warranted in future studies, that are well-powered a priori to detect potential mediation effects. This is described in the Discussion.

**Discussion**
• What do the authors mean by “original study” vs. sample on line 443?

We refer to the study for which the data were collected, that included individuals aged 15-49 (our study is restricted to individuals aged 15-24). To clarify, we have removed the term “original study” and rephrased two sentences to refer to the individuals who did not participate during data collection.

• I think that this study is also unique in that comparable data were collected for both females and males (see Weber, Ann M., et al. "Gender-related data missingness, imbalance and bias in global health surveys." BMJ global health 6.11 (2021): e007405).

We appreciate this point, and have added it to our Discussion section.

• An additional limitation is the use of logistic regression for a common outcome. Odds ratios will overestimate the risk ratio for outcomes exceeding about 10% prevalence.

While we do not dispute that odds ratios overestimate risk ratios when outcomes are not rare (i.e. when they exceed 10%), we do not see the use of logistic regression as a general limitation that compromises the utility of our findings. Odds ratios are a commonly reported metric in the social norms and family planning literature, and our use of them is in line with similar studies that have used logistic regression to report odds ratios, and have not mentioned this as a general limitation (e.g. Mejía-Guevara et al., (2020), Sedlander & Rimal (2019; 2021), Wegs et al., (2016), Komasawa et al., (2020), etc).

• Another limitation is that multiplicative interaction is of much less public health relevance than additive interaction (see VanderWeele, Tyler J., and Mirjam J. Knol. "A tutorial on interaction." Epidemiologic methods 3.1 (2014): 33-72.)

We very much appreciate this point and the reference. Our decision to use multiplicative interactions was to conduct analyses in line with the Expanded Theory of Normative Social Behavior (Rimal & Yilma, 2021) which uses multiplicative interactions. This is not to say, that additive interactions would not be a useful statistical complement to the theory’s propositions, but we chose our analyses guided by this theory, for which currently only multiplicative interactions have been applied. Further, the multiplicative interactions are tied to our hypothesis, particularly in how they would be falsified (i.e. by non-significant interaction terms, ostensibly from multiplicative interactions). However, this suggestion is very useful, and we will certainly be considering how to incorporate additive interactions in future work. In future interaction models, we will also consider how to specify a priori how hypotheses would be falsified if the results from multiplicative and additive interactions are different.

• The authors might be interested in the following reference for the need to measure gender norms: Weber, et.al. Gender norms and health: insights from global survey data. Lancet. 2019 Jun 15;393(10189):2455-2468. doi: 10.1016/S0140-6736(19)30765-2. Epub 2019 May 30. PMID: 31155273. Additionally, the case

We appreciate the reference and will consult it for future research. The reviewer’s comment cuts off in the last sentence.

**Overall**
• Be consistent with use of terms for participants. For example, in the paragraph starting on line 300, the authors refer to “women” and then “boys” (not “men”). I suggest using adolescent females (or girls) and adolescent males (or boys). While I assume the authors know the respondents’ sex but not their gender, they may prefer to use the terms “girls” and “boys” to emphasize their youth.

We appreciate this comment and have now used the terms male and female participants throughout.

• In order for to obtain an estimate of interaction on the additive scale (and to avoid other problems with logistic regression), the authors would need to use log-binomial or Poisson models to estimate relative risk or relative rate for a common outcome. I realize that this would be a big change, so am only informing the authors for future reference in terms of the limitations of logistic regression.

Thank you, this is very useful to know for future interaction models. Hopefully, the reviewer agrees with the point above (about sticking with the multiplicative model to align with extant literature), which would obviate the need to incorporate that point in this paper.

• Check for typos and grammatical errors.

Done.
