# Supplementary material for: The role of social norms on adolescent family planning in rural Kilifi county, Kenya
Source: PLoS One. 2023 Feb 2;18(2):e0275824. doi: 10.1371/journal.pone.0275824 (PMC9894424; doi:10.1371/journal.pone.0275824)
Supplement: Attachment — Submitted filename: Response to Reviewers .docx [file pone.0275824.s004.docx]

**Response to Reviewers**

August 26, 2022

Thank you for the opportunity to revise and resubmit our manuscript. We have made the two minor revisions requested by Reviewer #2. Comments from Reviewer #2 are in Black, and our responses are in Red.

**Comments from Reviewer #2**

 I wish to thank the authors for their consideration of my comments, which were addressed. I have two very minor suggestions, which I do not need to review:

* In the abstract, I still find the statement unclear: "...we estimated group-level normative influence by taking an average of an individual’s referents who use modern contraception." Could use text in author's response to a previous comment: "...by taking an average of referents’ modern contraception use."

Done.

* Line 168 at the end of the introduction: change "gender" to "sex" in :"we also stratified our analyses by gender and marital status"

Done.
